# Supplementary material for: Agave sisalana: towards distributed manufacturing of absorbent media for menstrual pads in semi-arid regions
Source: Commun Eng. 2023 Nov 30;2:81. doi: 10.1038/s44172-023-00130-y (PMC10955866; doi:10.1038/s44172-023-00130-y)
Supplement: Supplementary file 2 — Supplementary Information [file 44172_2023_130_MOESM2_ESM.pdf]

Supplementary information for

Agave Sisalana: towards distributed manufacturing of absorbent media for menstrual pads in semi-arid regions

October 25, 2023

Anton Molina[1,2,†], Anesta Kothari[2,†], Alex Odundo[3], Manu Prakash[2*]

[1]Department of Materials Science and Engineering Stanford University, 496 Lomita Mall, Stanford, California 94305

[2]Department of Bioengineering Stanford University, 443 Via Ortegs, Stanford, California 94305

[3]Olex TechnoEnterprises, Kisumu, Kenya

[*]To whom correspondence should be addressed; E-mail: manup@stanford.edu

[†] These authors contributed equally

This supplementary file includes:

- Supplementary Notes 1-8
- Supplementary Figures 1-7
- Supplementary Tables 1-9
- Supplementary Videos 1-3
- Supplementary References 1-36

Supplementary Note 1: Relationship between period poverty and resource availability

Data on preferred menstrual hygiene products (Fig. 1a) for select countries was obtained from Ref. [1] with original survey data collected between 2015-2017 (CC BY-ND 4.0). Over 500 million people are effected by period poverty, resulting in up to 20% non-participation in countries such as Sierra Leone. In 2014, up to 89.2 million girls dropped out of school. This number was derived from the total number of female dropouts (within menstruating ages) in lower secondary school (ages 12-14) and upper secondary school (ages 15-17) found in Ref. [2] (CC BY-SA 3.0 IGO).

The map on shown in Fig. 1b was generated from two datasets. The basemap was generated using Plotly Choloropleth map tool with GDP per capita data from the World Bank [3] (CC BY-4.0). This basemap was then superimposed with a semi-arid climate map extracted from Ref. [4] to show the geographic correlation between GDP per capita and areas where sisal can be cultivated and serve as a valuable commodity in resource-limited areas (CC BY-NC-SA 2.5). Data on global forest allocation was obtained from the FAO Global Forest Assessment 2020 report [5] (CC BY-NCSA 3.0 IGO).

There is an uncorrelated relationship between GDP per capita and imports of menstrual products (normalized by the total number of females between the ages of 15 and 49) as shown in Supplemental Fig. 1. Low import values, as seen in Japan and Bangladesh, may be attributed to either high domestic production of menstrual products or its population is resorting to other means of menstrual hygiene management (i.e. cloth rags). In contrast, countries, such as Germany, have high import values, suggest these countries resorting more to imports rather than domestically-produced products. Kiribati is an anomaly where import value is high despite an extremely low GDP per capita.

Supplementary Note 2: Rhelogical characterization of viscous test liquid

The viscosity of the test liquid was measured using a rheometer (TA Instruments ARES-G2) at 30 °C and 37 °C, using a Couette geometry (DIN Bob: 27.671 mm diameter, 41.59 mm 694 length; Cup, 29.986 mm diameter, anodized aluminum). The temperature of the samples were equilibrated for 2 minutes before the start of the experiments. Samples were tested in flow sweep, with shear rate ramped up and down from 1 s^{-1} to 100 s^{-1}. Measurements were repeated in triplicate. The visocity at 30°C and 37°C is measured to be ∼4.2 cP and ∼3.5 cP, respectively. The sampels did not display any shear thinning or hysteresis. This is comparable to real blood which has viscosity ∼3.5-5.5 cP [6].

Supplementary Note 3: Mild delignification of decorticated sisal fibers

Fibers are cut into short segments (∼10 mm long) and boiled in water to remove any water soluble components. Following the procedure described in Ref. [7], the stems were then delignified using 10% (v/v) peroxyformic acid (synthesized in situ by combining 30% hydrogen peroxide with 95% formic acid in a 1 : 1 mole-to-mole ratio using 1% (v/v) sulfuric acid as a catalyst[8]) at 50°C overnight. The fibers were then treated with 4% (w/v) sodium hydroxide for 2 hours at 50°C. Finally, the fibers were washed with three times with equivalent volumes of deionized water or until the pH was neutral. It is important to note that the fibers were subject to approximately of 30 s of vigorous shaking during each wash. This introduction of mechanical energy is analogous to the use of Hollander beaters used in conventional paper making and is important for defibrilating the fibers 100 μm macrofibrillar bundles into smaller microfibers.

In another realization, we adapted the procedure described in Ref. [9] which involves the use of Fe(II) as a catalyst to initialize the decomposition of peroxyformic acid into reactive

radical species . Here, cut fibers were delignified using 1% (v/v) peroxyformic acid. In this instance, the amount of sulfuric acid was increased to 4% in order to accelerate the conversion of formic acid and hydrogen peroxide into peroxyformic acid [10, 11, 12]. The mixture was left to react for 45 min. before adding 400 μL of a solution containing $Fe_2(SO_4)_3$ (1 kg/L). This mixture was then left to react overnight at 50°C overnight. The fibers were then treated with sodium hydroxide and washed as described above.

To compare with typical delignification methods used for processing non-wood fibers, we followed the soda pulping process described in Ref. [13]. Here, 5g of cut and dried fibers were combined with 400 mL of 8% (w/w) sodium hydroxide at 80-90°C for 3 hours. The fibers were then washed with deionized water as described above.

Supplementary Note 4: Life-cycle analysis of carbon footprint for sisal cellulose microfibers

We analyze the two different life-cycle analysis (LCA) carbon footprint scenarios for the production of sisal cellulose microfibers corresponding to the lab scale procedure carried out in this work and a aspirational scenario assuming the on-site generation of key reagents using commercially available technologies. The life cycle inventory (LCI) for the sisal cellulose microfibers produced according to the lab scale and on-site scenario are detailed in Table 1 and 2, respectively. The system boundary is cradle-to-gate and includes the cultivation and harvesting of raw materials, transportation, and processing. Unless, otherwise stated, the LCI data of upstream production of chemicals, deionized water, and electricity was collected from EcoInvent 3.01 database.

In the labs scale scenario, energy was assumed to be a US electricity mix (Western Electricity Coordinating Council) used for the processing of sisal fibers and for all upstream chemical production. The greenhouse gas emissions generated from cultivation and harvest of sisal was set to 0.17 kg CO2-eq/kg raw sisal [14], since it better reflects the carbon footprint associated with dry decortication. Chemicals were assumed to be transported a distance

of 352 km based on the average distances of truck transportation of basic chemicals in the United States in 2017 [7]. Sisal is assumed to be transported 222 km based on the average distances of truck transportation of logs and other wood in rough in the United States in 2017 [7]. The truck was assumed to be a freight truck (16-32 metric ton). The carbon footprint is reported in kg CO2-eq using 100 year global warming potential conversion factors reported by the 2007 Intergovernmental Panel on Climate Change [15]. The carbon sequestration of the processed biomass was not included in our calculation since absorbent materials are expected to have a short lifetime, minimizing any impact of sequestration.

Evaluation of the on-site production scenario is based on information collected on commercially available systems from the literature and are presented in Table 2. Production of hydrogen peroxide was based on the exposure-surface solid polymer electrolyte system [16] currently being commercialized [17]. Production of formic acid was based on the study by Lust *et al* where they used a micro flow electrolysis cell based on a commercially available RuCl2[PPh3] (Dichloro- tristriphenylphosphin-ruthenium(II)) catalyst operated at 50°C and atmospheric pressure [18]. Prior work has shown that adequate quantities of peroxyformic acid can be produced on-demand, at scale, and in a safe manner given the availability of hydrogen peroxide and formic acid [19, 20, 21]. The life cycle inventories and transport of sulfuric acid, sodium hydroxide, and iron sulfate were taken to be the same as in the lab-scale scenario. The electricity used to drive the on-site production of reagents was assumed to come from renewable solar energy and have a negligible carbon footprint.

Supplementary Note 5: Life cycle carbon analysis for sisal cellulose microfibers by activity

Estimates for the contribution of harvesting and cultivation activities within the cradle-to-gate scope are collected in Table 3. Forestry activities associated with the production of softwood timber is estimated to be 0.075 kg CO2-eq/kg softwood fluff pulp [22]. The cultivation and harvesting associated with cotton production is estimated at 4.38 kg CO2-

eq/kg cotton [23], with the majority of contribution coming from the upstream production of fertilizers. The cultivation and harvesting associated with sisal is estimated at 0.17-0.66 kg CO2-eq/kg. The largest contributor is due to methane generation of decortication waste when high-throughput wet decorticators are employed [14].

Estimates for the contribution of processing activities within the cradle-to-gate scope are collected in Table 4. The processing of softwood amounts to 0.984 kg CO2-eq/kg softwood fluff pulp [22]. The processing of cotton amounts to 0.575 kg CO2-eq/kg bleached cotton [23]. The details of sisal processing are described in Supplemental Note 4 and amount to 0.86-3.1 kg CO2-eq/kg sisal fluff pulp.

Estimates for the contribution of transportation activities within the cradle-to-gate scope are collected in Table 5. Transportation of raw timber materials can vary significantly. Typical collection radii vary from 100-550 km [24, 25], though the majority fall within the range 100-200 km [24]. This corresponds to 0.065-0.13 kg CO2-eq/kg fluff pulp, emphasizing the importance of localizing raw material sourcing for the production of bleached softwood fluff pulp [22]. For the production of bleached cotton, a carbon footprint associated with transportation has been reported to be 0.0241-0.0315 kg CO2-eq/kg cotton [23].

Supplementary Note 6: Life-cycle carbnfootprint analysis for post-gate scope

We defined a post-gate scope that accounts for the transportation of the absorbent material beyond its production. This includes transportation of fluff pulp to a pad manufacturing facility and transportation of the assembled product to the final market. Given the complexity of the supply chains involved, this is difficult to estimate and so we consider three scenarios described in the literature and ignore last mile distribution. The transportation mixes are described in Tables 6, 7, and 8.

In one scenario [26], a conventional sanitary pad manufacturer reports that transportation of raw material to their manufacturing facility and subsequent delivery of the finished product

to consumers consists of ship (2048 km), truck (1342 km), and rail (141 km). Inbound raw materials are transported primarily by sea freight, whereas outbound finished products are transported by road and rail . This totals 3532 km and represents a 0.275 kg CO2-eq/kg embodied fluff pulp.

In a second scenario [26], considering the manufacture of sanitary pads from bamboo-derived fluff pulp, bamboo-derived fluff pulp is transported by truck (180 km) to a river port. A barge travels to an international port (3572 km) for international shipping. In this scenario, the final product is shipped to Gothenberg, Sweden through the Suez Canal (18,875 km) and then by truck to a distribution facility (170 km). Last mile distribution is not considered in this scenario. This totals 22,797 km and represents a 0.467 kg CO2-eq/kg embodied fluff pulp.

In a third scenario [27], pads are shipped from Sweden to the port in Mombasa, Kenya (10,793 km) and then by truck to Kempala, Uganda (1140 km). In this case, transport of fluff pulp to the pad manufacturing facility is not considered. This totals, 11,933 km and represents a 0.321 kg CO2-eq/kg embodied fluff pulp.

Supplementary Note 7: Water consumption analysis for fluff processing

We compare the water consumption of our process two processing scenarios to three alternatives: 1) fluff pulp production[28, 22], 2) bleached cotton[29, 23], and 3) a medium-sized pad manufacturing facility operating in a low resource environment [27]. Estimates for water consumption associated with alternative processes are collected from the literature and presented in Table 9. What follows is a brief discussion to contextualize the tabulated data.

Freshwater consumption in a typical pulp and paper mill ranges from 9-90 kg H2O/kg fluff pulp, with the average estimated to be $\sim$ 30 kg H2O/kg fluff pulp, with the bleaching process associated with pulp production representing one of the largest contributors to freshwater consumption [28]. A detailed life cycle inventory for the production of bleached softwood

fluff pulp estimates the water consumption at 61.78 kg/kg fluff pulp [22].

Water consumption for bleaching of cotton was obtained from life cycle inventories associated with a case study data describing two textile facilities in Italy and China which use 367 and 85 kg H2O/kg for dyed and bleached cotton, respectively [29].

Musazi *et al.* describe a facility engaged in the production of disposable menstrual pads in rural Uganda using locally sourced materials. This facility consumes $\sim$ 3000 L/day to produce 2741 pads. The dimensions of a pad are 5x20 cm. Assuming a density of 0.05 g/cm^3, we estimate that each pad contains $\sim$ 5 g absorbent material which is consistent with CMPs we have examined in our lab. We therefore establish upper and lower limits on water consumption based on 4-8g per pad giving 273-136 kg H2O/kg absorbent material.

Supplementary Note 8: Description of water catchment area requirements in semi-arid regions

Another consideration is the amount of water that can be accessed in arid and semi-arid regions. Arid climates constitute the largest climate type by land surface area (40%) and are characterized by a precipitation P between 250-500 mm/year [30]. We estimate the catchment area A_c required for the on-site fiber extraction described in this work:

$$A_c = \frac{V_c}{PC} \tag{1}$$

where V_c is the volume of rain water harvested, and C is the non-dimensional run-off coefficient [31]. We take C to be 0.8 which is characteristic for corrugated metal roofs [32]. Assuming production of 3000 pads/day, requiring daily production of 15 kg fluff pulp, annual water demand would between 244,185-763,763 kg H20. Using the boundaries for rainfall in semi-arid climates, we can estimate $A_c = 610 - 1,909m^2$.

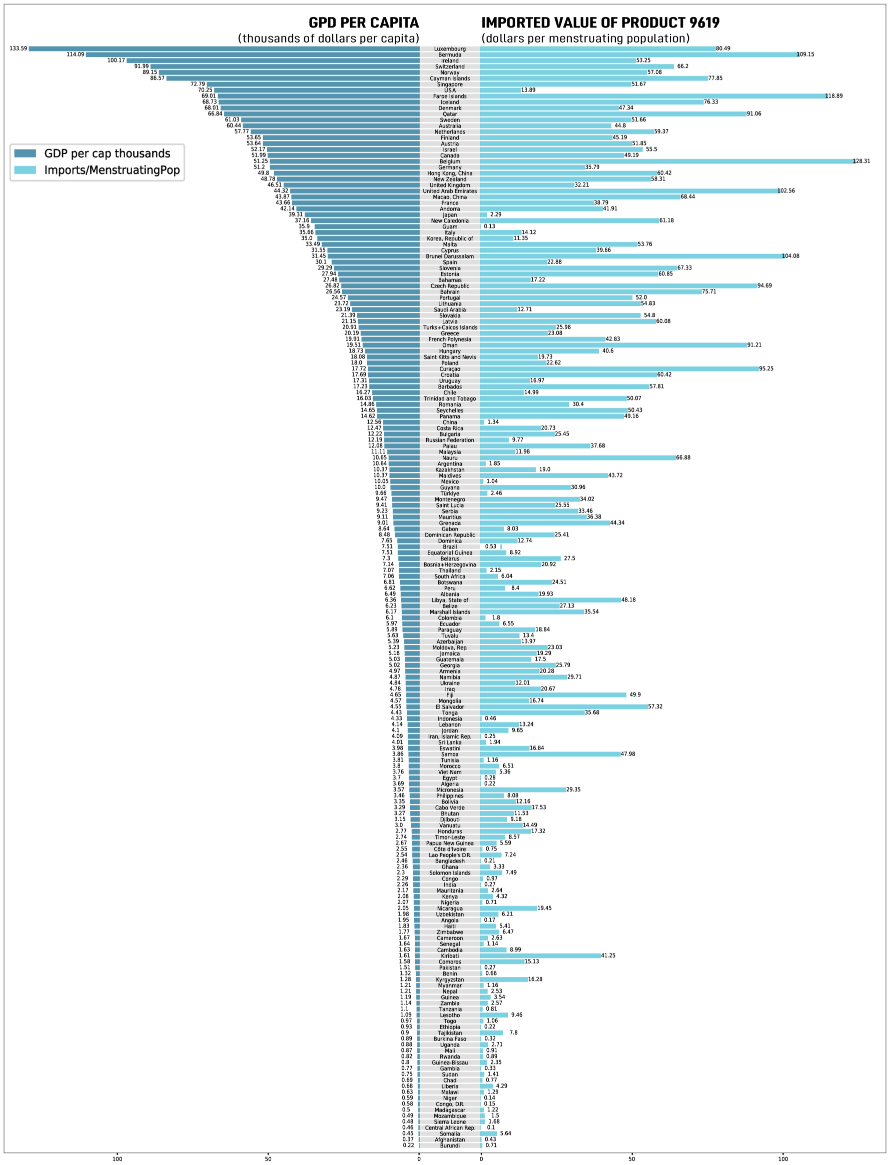

Supplementary Figure 1: Mirrored bar chart comparing GDP per capita with the import value of product 9619 (sanitary towels (pads) and tampons, napkins and napkin liners for babies and similar articles, of any material) normalized by total menstruating population (females ages 15-49). GDP per capita was taken directly from The World Bank [3]. Import value data was obtained from the International Trade Centre [33]. Menstruating population was derived from the total population [34], percent of female population [35], and percent of menstruating females [36].

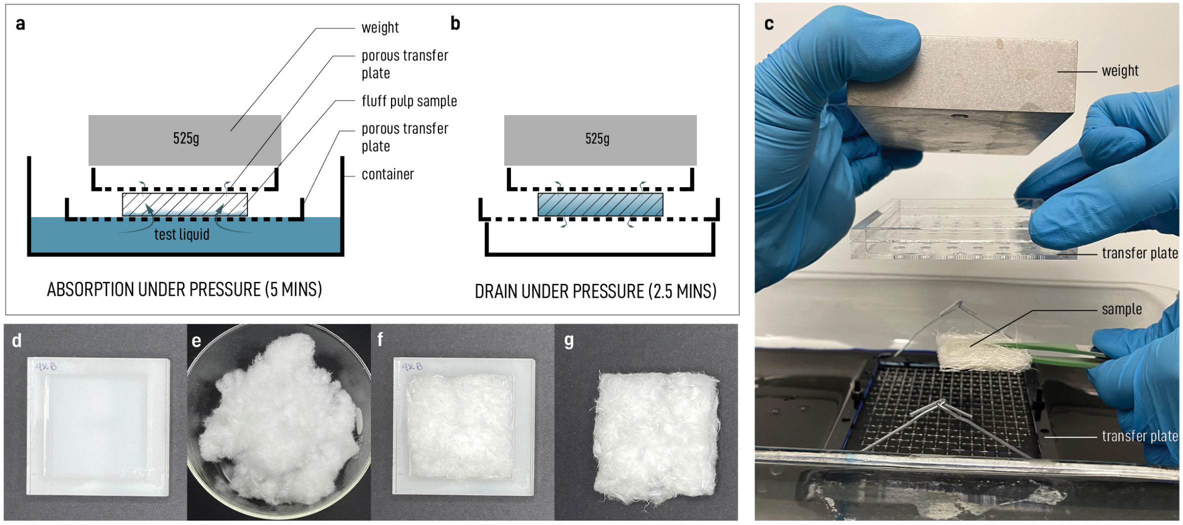

Supplementary Figure 2: Absorption under pressure (AUP) Schematic diagram of AUP setup during liquid uptake [a] and draining [b]. [c] Photograph of setup described in [a-b]. [d-g] Photographs showing preparation of test square from blended fluff pulp using acrylic mold.

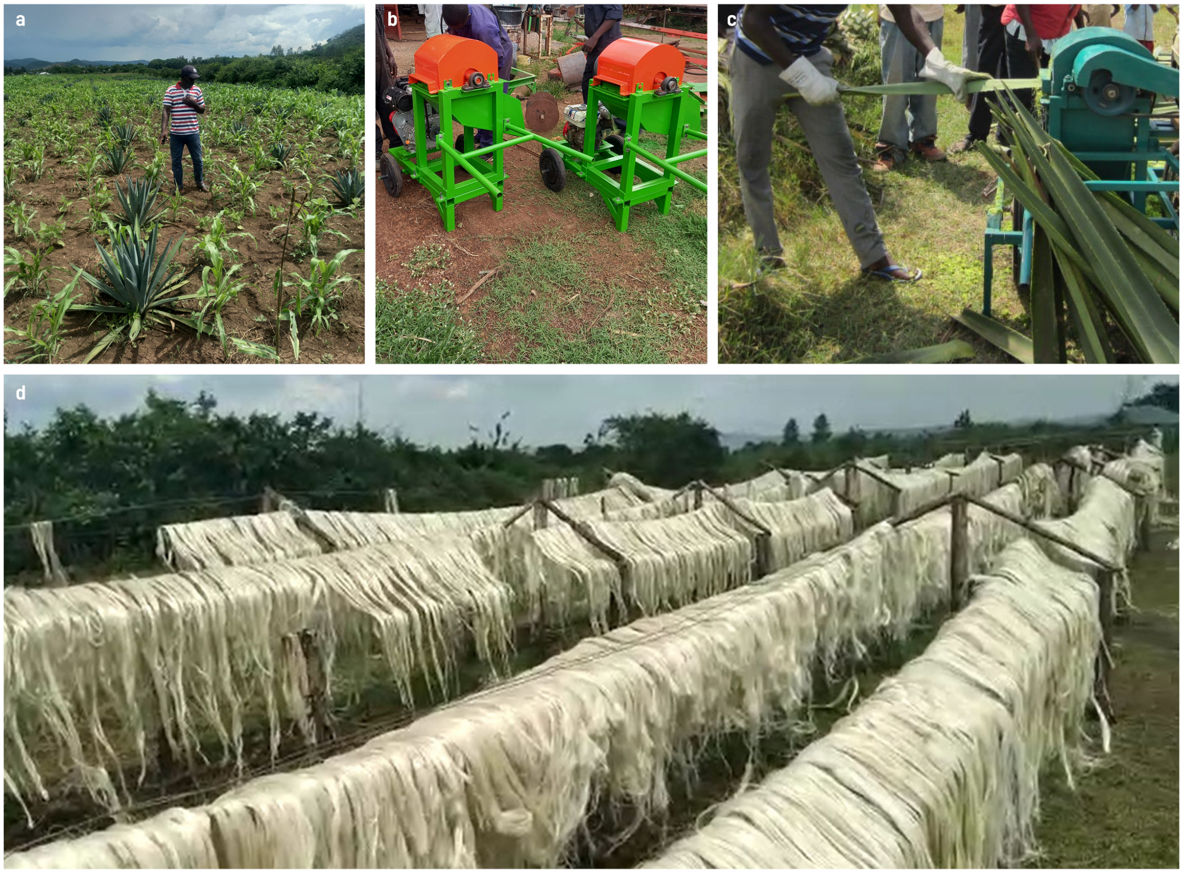

Supplementary Figure 3: Sisal cultivation, harvest, and decortication on the field in Kisumu, Kenya [a] Sustainable cultivation of sisal. Intercroping of sisal with complementary crops such as maize and legumes reduces the effects of soil depletion. [b] Manually-operated sisal decorticators. [c] Operation of decorticator shown in [b]. [d] Decorticated fibers being dried in the field.

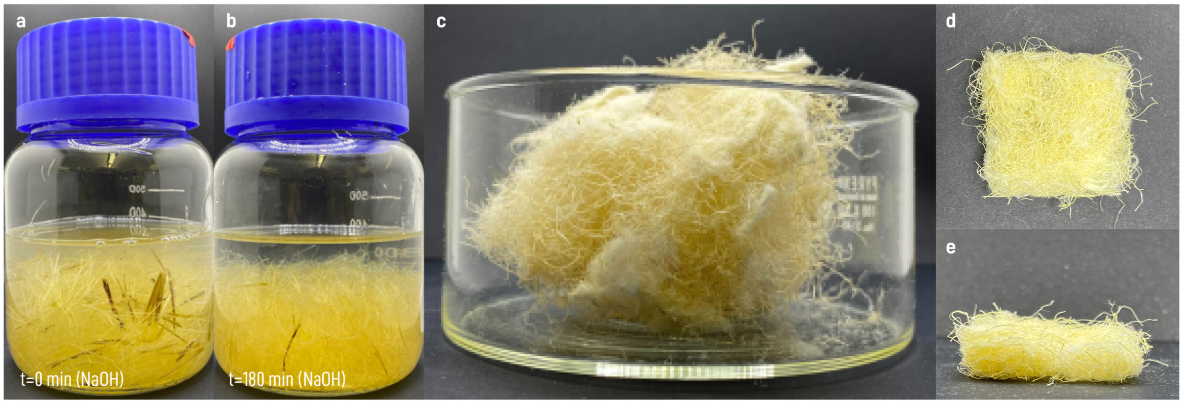

Soda Pulping
8% NaOH (85°C 3hrs)

Supplementary Figure 4: Soda pulping Boiled sisal macrofibers before [a] and after 3h in 8% sodium hydroxide at 85°C [b]. [c] Photograph of fibers from [b] after air drying and blending. Photograph of top [d] and cross section [e] view of test square prepared from material in [c].

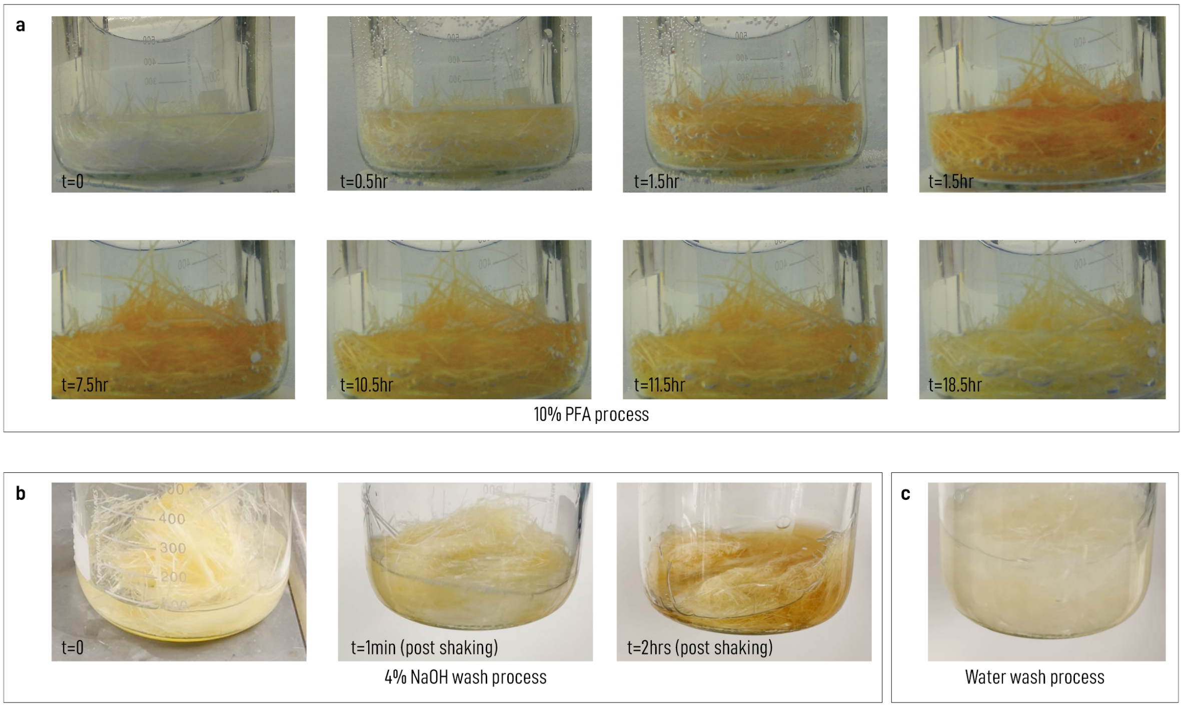

Supplementary Figure 5: Time series showing visible effects of chemical treatment and washes. [a] 10% peroxyformic acid treatment over 18.5 hours in 50°C. The orange color at 5h indicates fraction of lignin released into solution. Disappearance of orange color at end of reaction indicates decomposition of lignin fraction in solution. [b] 4% sodium hydroxide wash over 2 hours in 50°C. The orange color at 2h indicates a solubilization of remaining lignin. The liquid containing lignin and hemicellulose is then removed. [c] The water wash removes residual chemicals and helps to debundle the delignified material into cellulose microfibers.

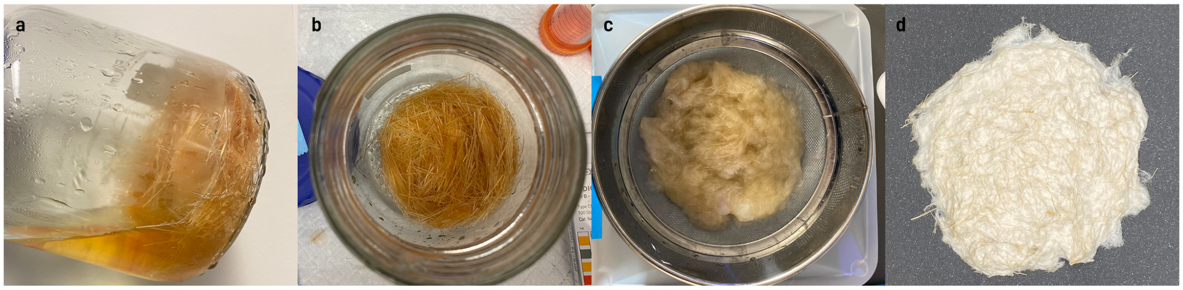

Supplementary Figure 6: Reducing peroxyformic acid concentration [a-b] 1% peroxyformic acid activated with Fe(II) after 18h at 50°C. Orange color in solution indicates lignin fraction in solution is not fully decomposed. [c] 4% sodium hydroxide wash and subsequent water washes and left to air dry yielding a fluff board [d].

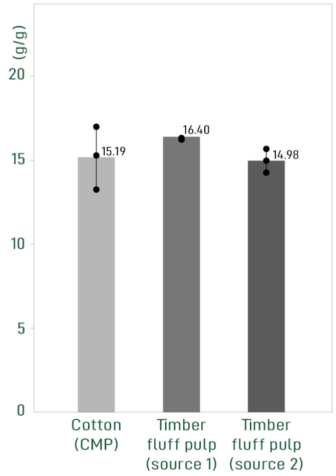

Supplementary Figure 7: Baseline absorption Bar chart comparing the absorption capacities of cotton CMP with timber fluff pulp obtained from two sources that are used in commercially-available-pads. Error bars correspond to one standard deviation.

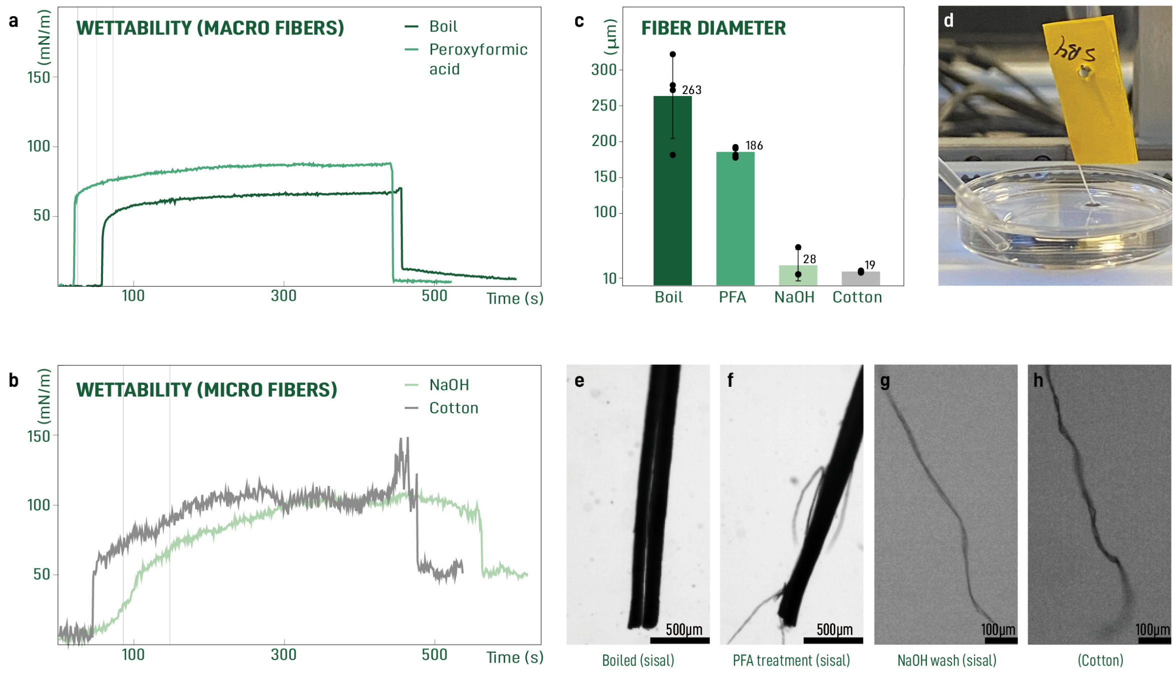

Supplementary Figure 8: Static contact angle tensiometry Representative data showing wettability versus time for single macrofibers [a] and microfibers [b]. [c] Diameters of fibers used to calculate contact angle from wettability data in [a-b]. Error bars correspond to one standard deviation. [d] Photo of experimental setup. Representative light microscopy images of single macrofibers [d-e] and microfibers [f-g].

Supplementary Table 1: Input and output (life cycle inventory) of the sisal processing

Input/Output	Quantity	Unit	Model
Step 1: pretreatment (boil step)			
Input			
Cultivation, harvest, decortication of sisal	1.667	kg	[14]
Transport of decorticated fibers to fluffing plant	0.567	tkm	Transport, lorry 3.5-16t, fleet average/RER (from sisal LCA)
Energy	0.16	kWh	Electricity, low voltage WECC US ONLY market for — Alloc Def S [7]
Water	25	kg	Water deionized from tap water at user GLO market for, Alloc Def S
Output			
Pre-treated sisal fibers	1.667	kg	
Step 2: treatment with peroxyformic acid solution			
Input			
Pre-treated sisal fibers	1.667	kg	
Energy	1.92	kWh	Electricity, low voltage WECC US ONLY market for — Alloc Def S [7]
Formic acid	0.475	kg	Formic acid RoW oxidation of butane — alloc def S
Hydrogen peroxide	0.165	kg	Hydrogen peroxide, without water 50% in solution state RoW Alloc Def S
Sulfuric acid	0.25	kg	Sulfuric acid RoW — Alloc Def S
Water	20.68	kg	Water deionized from tap water at user GLO market for, Alloc Def S
Iron sulfate	0.089	kg	Iron sulfate GLO — market for — Alloc Def, S
Transport of chemicals	0.346	tkm	PFA - Transport, freight, lorry 16-32 metric ton, EURO3 RER — transport, lorry 16-32 metric ton, EURO3 — Alloc Def, S
Output			
Treated sisal fibers	1.667	kg	

Table continued on next page.

Supplementary Table 1: (continued) Input and output (life cycle inventory) of the sisal processing

Input/Output	Quantity	Unit	Model
Step 3: sodium hydroxide wash			
Input			
Treated sisal fibers	1.667	kg	
Energy	0.32	kWh	Electricity, low voltage WECC US ONLY market for — Alloc Def S [7]
Sodium hydroxide	2.167	kg	Sodium hydroxide, without water, in 50% solution state GLO
Water	23.92	kg	Water deionized from tap water at user GLO market for, Alloc Def S
Transport of chemicals	0.765	tkm	PFA - Transport, freight, lorry 16-32 metric ton, EURO3 RER — transport, lorry 16-32 metric ton, EURO3 — Alloc Def, S
Output			
Alkali-washed sisal fibers	1.0	kg	
Step 4: water wash			
Input			
Alkali-washed sisal fibers	1.667	kg	
Water	50	kg	Water deionized from tap water at user GLO market for, Alloc Def S
Output			
Wet pulp (not including absorbed water)	1.0	kg	
Step 5: fluffing			
Input			
Dry fluff board	1.0	kg	
Energy	0.067	kWh	Electricity, low voltage WECC US ONLY market for — Alloc Def S [7]
Output			
Dry pulp	1.0	kg	

Supplementary Table 2: Life cycle inventory (onsite production)

Input/Output	Quantity	Unit	Model
Onsite production of hydrogen peroxide			
Input			
Energy	3.12	kWh	Renewable energy
Water	16.5	kg	Water deionized from tap water at user GLO market for, Alloc Def S
Output			
Hydrogen peroxide	0.165 [17]	kg	[17]
Onsite production of formic acid			
Input			
Energy	7.12	kWh	Renewable energy
Water	3.4	kg	Water deionized from tap water at user GLO market for, Alloc Def S
Output			
Formic acid	0.475	kg	[18]

Supplementary Table 3: Harvest and Cultivation

Materials	Carbon Footprint (CO_2eq)
Sisal	0.17-0.66 kg (inclusive of transport of raw materials) [14]
Timber	0.075 kg [22]
Cotton	2.93 kg (cultivation) [23], 1.45 kg (harvest) [29]

Supplementary Table 4: Fluff Processing

Materials	Carbon Footprint (CO_2eq)
Sisal	0.86-3.10 kg
Timber	0.984 kg [22]
Cotton	0.29 kg (bleaching), 0.28 kg (combing), 0.0052 kg (fiber production) [29]

Supplementary Table 5: Transport of Raw Fibers

Materials	Carbon Footprint (CO_2eq)
Sisal	0.0028 kg [14]
Timber	0.065-0.13 kg [22]
Cotton	0.0241-0.0315 kg [23]

Supplementary Table 6: Post gate transportation - Scenario 1 - each distance assumption was taken from [26]

Transport	Quantity	Unit	Footprint	Unit	Model
Sea	2.048 [26]	tkm	0.0125	CO_2eq	Transport, freight, sea, transoceanic ship GLO — processing — Alloc Def, S
Road	1.342 [26]	tkm	0.256	CO_2eq	Transport, freight, lorry 16-32 metric ton, EURO4 RoW — transport, freight, Alloc Def S
Rail	0.141 [26]	tkm	0.00647	CO_2eq	Transport, freight train CN — diesel — Alloc Def, S
Total	3.532	tkm	0.275	CO_2eq	

Supplementary Table 7: Post gate transportation - Scenario 2 - each distance assumption was taken from [26]

Transport	Quantity	Unit	Footprint	Unit	Model
Sea	18.875 [26]	tkm	0.218	CO_2eq	Transport, freight, sea, transoceanic ship GLO — processing — Alloc Def S
Road	0.170 [26]	tkm	0.0603	CO_2eq	Transport, freight, lorry 16-32 metric ton, EURO4 RoW — transport, freight, Alloc Def S
River barge	3.572 [26]	tkm	0.189	CO_2eq	Transport, freight, inland waterways, barge GLO market for, Alloc Def S
Total	22.797	tkm	0.467	CO_2eq	

Supplementary Table 8: Post gate transportation - Scenario 3 - each distance assumption was taken from [27]

Transport	Quantity	Unit	Footprint	Unit	Model
Sea	10.793 [27]	tkm	0.125	CO_2eq	Transport, freight, sea, transoceanic ship GLO — processing — Alloc Def S
Road	1.140 [27]	tkm	0.196	CO_2eq	Transport, freight, lorry 16-32 metric ton, EURO4 RoW — transport, freight, Alloc Def S
Total	11.933	tkm	0.321	CO_2eq	

Supplementary Table 9: Water consumption inventory

Materials	Water footprint (kg)
This work (lab scale)	119.6 (no recycling); 44.6 (with recycling)
This works (with onsite production)	139.5 (no recycling); 64.5 (with recycling)
Bleached softwood fluff pulp	61.78 [22]; 9-90 (average 60) [28]
Bleached cotton	85-362 [29]; 1380-2100 [23]
Papyrus	91-218.9 [27]

Supplementary Video 1: Measurement of absorption under pressure.

Supplementary Video 2: Single-head sisal decorticator in use.

Supplementary Video 3: Fluffing process following treatment with peroxyformic acid.

Supplementary References

[1] Amaya, L., Marcatili, J. & Bhavaraju, N. *Advancing Gender Equity by Improving Menstrual Health: Opportunities in Menstrual Health and Hygiene* (FSG, 2020).

[2] Global Education Monitoring Report Team, U. I. f. S. Leaving no one behind: How far on the way to universal primary and secondary education? *Global Education Monitoring Repor* (2016). URL `https://unesdoc.unesco.org/ark:/48223/pf0000245238`.

[3] The World Bank. *GDP per capita (current US$)* (2023). URL `https://data.worldbank.org/indicator/NY.GDP.PCAP.CD`.

[4] Peel, M. C., Finlayson, B. L. & McMahon, T. A. Updated world map of the köppen-geiger climate classification. *Hydrology and Earth System Sciences* **11**, 1633–1644 (2007).

[5] FAO. Global forest resources assessment 2020: Main report (2020).

[6] Nader, E. & et al. Blood rheology: Key parameters, impact on blood flow, role in sickle cell disease and effects of exercise. *Frontiers in Physiology* **10** (2019).

[7] Li, Z. & et al. Sustainable high-strength macrofibres extracted from natural bamboo. *Nature Sustainability* **5**, 235–244 (2021).

[8] Greenspan, F. P. The convenient preparation of per-acids. *Journal of the American Chemical Society* **68**, 907–249074 (1946).

[9] Haverty, D., Dussan, K., Piterina, A. V., Leahy, J. J. & Hayes, M. H. B. Autothermal, single-stage, performic acid pretreatment of miscanthus x giganteus for the rapid fractionation of its biomass components into a lignin/hemicellulose-rich liquor and a cellulase-digestible pulp. *Bioresource Technology* **109**, 173–177 (2012).

[10] Santacesaria, E., Russo, V., Tesser, R., Turco, R. & Di Serio, M. Kinetics of performic acid synthesis and decomposition. *Industrial Engineering Chemistry Research* **56**, 12940–12952 (2017).

[11] Sawaki, Y. & Ogata, Y. The kinetics of the acid-catalyzed formation of peracetic acid from acetic acid and hydrogen peroxide. *Bulletin of the Chemical Society of Japan* **75**, 2103–2106 (1965).

[12] Dul'neva, L. V. & Moskvin, A. V. Kinetics of formation of peroxyacetic acid. *Russian Journal of General Chemistry* **75**, 1125–1130 (2005).

[13] Scharpf, E. *et al.* Highly absorbent and retentive fiber material (U.S. Patent 9365972B2, 2014).

[14] Broeren, M. L. M. & et al. Life cycle assessment of sisal fibre – exploring how local practices can influence environmental performance. *Journal of Cleaner Production* **149**, 818–827 (2017).

[15] IPCC AR4 Climate Change. *The physical science basis: summary for policymakers. Contribution of working group I to the fourth assessment report of the intergovernmental panel on climate change.* (2007).

[16] Yamanaka, I. & Murayama, T. Neutral h2o2 synthesis by electrolysis of water and o2. *Angewandte Chemie International Edition* **47**, 1900–1902 (2008).

[17] HP Now. *Technical Datasheet: HP Gen A series* (2022).

[18] Lust, D. *et al.* Decentralized city district hydrogen storage system based on the electrochemical reduction of carbon dioxide to formate. In *Proceedings of the 13th International Renewable Energy Storage Conference 2019 (IRES 2019)*, 137–144 (Atlantis Press, 2019/11). URL https://www.atlantis-press.com/article/125923326.

[19] Ebrahimi, F., Kolehmainen, E., Oinas, V., P. andHietapelto & Turunen, I. Production of unstable percarboxylic acids in a microstructured reactor. *Chemical Engineering Journal* **167**, 713–717 (2011).

[20] Gaikwad, S. M. & et al. Process intensification for continuous synthesis of performic acid using corning advanced-flow reactors. *Green Processing and Synthesis* **6** (2017).

[21] Ebrahimi, F., Kolehmainen, E. & Turunen, I. Safety advantages of on-site microprocesses. *Organic Process Research Development* **13**, 965–969 (2009).

[22] Buitrago-Tello, R., Venditti, R. A., Jameel, H., Yao, Y. & Echeverria, D. Carbon footprint of bleached softwood fluff pulp: Detailed process simulation and environmental life cycle assessment to understand carbon emissions. *ACS Sustainable Chemistry Engineering* **10**, 9029–9040 (2022).

[23] Vilabrille Paz, C., Ciroth, A., Mitra, A., Birnbach, M. & Wunsch, N. *Comparative life cycle assessment of menstrual products* (GreenDelta GmbH, commissioned by einhorn products GmbH, 2020).

[24] Rasmussen, E. *A summary of EBRD's new Russian forest policy dialogue study.* (Northern Dimension Business Council, 2012). URL http://www.ebrd.com/downloads/news/120329pres.pdf.

[25] Börjesson, M. H. & Ahlgren, E. O. *Technology Brief I07: Pulp and Paper Industry* (International Energy Agency - Energy Technology Systems Analysis Program, 2015). URL https://iea-etsap.org/E-TechDS/PDF/I07$_{P}ulpPaper_{M}ay2015OK.pdf$.

[26] Mirzaie, A. *Life cycle assessment of the unbleached bamboo sanitary pad: A case study performed at Hempur* (Degree project in Industrial Ecology: Second Cycle, School of Architecture and Built Environment, KTH Royal Institute of Technology, Department of Sustainable Development, Environmental Science and Engineering, 2021).

[27] Musaazi, M. K. & et al. Quantification of social equity in life cycle assessment for increased sustainable production of sanitary products in uganda. *Journal of Cleaner Production* **96**, 569–579 (2015).

[28] Echeverria, D., Venditti, R., Jameel, H. & Yao, Y. A general life cycle assessment framework for sustainable bleaching: A case study of peracetic acid bleaching of wood pulp. *Journal of Cleaner Production* **290**, 125854 (2021).

[29] Cardoso, A. A. M. *Life cycle assessment of two textile products: wool and cotton* (Thesis: S.M., Faculty of Engineering, Porto University, Department of Chemical Engineering, 2013).

[30] Lane, L. J. & Nichols, M. H. Semi-arid climates and terrain. *Encyclopedia of Earth Science* 556–558 (1999).

[31] Thomas, T. H. & Martinson, D. B. *Roofwater harvesting : a handbook for practitioners* (IRC International Water and Sanitation Centre, Delft, The Netherlands, 2007).

[32] Biswas, B. K. & Mandal, B. H. Construction and evaluation of rainwater harvesting system for domestic use in a remote and rural area of khulna, bangladesh. *International Scholarly Research Notices* 1–6 (2014).

[33] International Trade Centre. *List of importers for the selected product. Product: 9619 Sanitary towels (pads) and tampons, napkins and napkin liners for babies, and similar articles, of any material* (2019).

[34] The World Bank. *Population, total* (2023). URL `https://data.worldbank.org/indicator/SP.POP.TOTL`.

[35] The World Bank. *Population, female (% of total population)* (2023). URL `https://data.worldbank.org/indicator/SP.POP.TOTL.FE.ZS`.

[36] The World Bank. *Population ages 15-64, female (% of female population)* (2023). URL https://data.worldbank.org/indicator/SP.POP.1564.FE.ZS.
