# Supplementary material for: Agave sisalana: towards distributed manufacturing of absorbent media for menstrual pads in semi-arid regions
Source: Commun Eng. 2023 Nov 30;2:81. doi: 10.1038/s44172-023-00130-y (PMC10955866; doi:10.1038/s44172-023-00130-y)
Supplement: Supplementary file 3 — Description of Additional Supplementary Files [file 44172_2023_130_MOESM3_ESM.pdf]

Description of Additional Supplementary Files

File name: Supplementary Video 1

Description: Single-head sisal decorticator in use.

File name: Supplementary Video 2

Description: Measurement of absorption under pressure (AUP)

File name: Supplementary Video 3

Description: Fluffing process following treatment with peroxyformic acid.
